# Chronic Myeloid Leukemia: Part II—Cost of Care Among Patients in Advanced Phases or Later Lines of Therapy in Chronic Phase in the United States from a Commercial Perspective

**DOI:** 10.36469/001c.36976

**Published:** 2022-08-04

**Authors:** Ehab L. Atallah, Rodrigo Maegawa, Dominick Latremouille-Viau, Carmine Rossi, Annie Guérin

**Affiliations:** 1 Medical College of Wisconsin, Milwaukee; 2 Novartis Pharmaceuticals Corporation, East Hanover, New Jersey; 3 Analysis Group, Inc, Montréal, Québec, Canada

**Keywords:** accelerated phase/blast crisis, chronic myeloid leukemia, cost of care, fourth-line therapy, healthcare resource utilization, third-line therapy

## Abstract

**Background:** Tyrosine kinase inhibitors (TKIs) are the standard-of-care treatment for chronic myeloid leukemia in chronic phase (CML-CP). Despite advances in therapy, there remains a proportion of patients with CML-CP that are refractory/intolerant to TKIs, and these patients cycle through multiple lines of therapy. Moreover, even with TKIs, some patients progress to accelerated phase/blast crisis (AP/BC), which is associated with particularly poor clinical outcomes.

**Objectives:** To describe real-world treatment patterns, healthcare resource utilization (HRU), and costs of patients with CML-CP reaching later lines of therapy or progressing to AP/BC in the United States.

**Methods:** Adult CML patients from administrative claims data (January 1, 2000–June 30, 2019) were classified by health state: on third-line (CML-CP On Treatment), on fourth or later lines (CML-CP Post-Discontinuation), or progressed to AP/BC (CML-AP/BC). Outcomes were assessed by health state.

**Results:** There were 296 (4620 patient-months), 83 (1644 patient-months), and 949 (25 593 patient-months) patients classified in the CML-CP On Treatment, CML-CP Post-Discontinuation, and CML-AP/BC cohorts, respectively. Second-generation TKIs (nilotinib, dasatinib, and bosutinib) were most commonly used in the CML-CP On Treatment (69.1% of patient-months) and CML-CP Post-Discontinuation cohorts (59.1% of patient-months). Three-month outpatient incidence rates (IRs) were 7.6, 8.3, and 7.0 visits in the CML-CP On Treatment, CML-CP Post-Discontinuation, and CML-AP/BC cohort, respectively, with mean costs of 597perservice.Three−monthinpatientIRswere0.6,0.7,and1.4daysintheCML−CPOnTreatment,CML−CPPost−Discontinuation,andCML−AP/BCcohort,respectively,withmeancostsof5892 per day. Mean hematopoietic stem cell transplantation cost was 352 333;mean3−monthterminalcarecostwas107 013.

**Discussion:** Cost of CML care is substantial among patients with CML reaching third-line, fourth or later lines, or progressing to AP/BC, suggesting that the disease is associated with a significant economic and clinical burden. From third-line to fourth or later lines, HRU was observed to increase, and the incidence of inpatient days was particularly high for those who progressed to AP/BC.

**Conclusion:** In this study, patients with CML cycling through TKIs in later lines of therapy or progressing to AP/BC experienced substantial HRU and costs, suggesting unmet treatment needs.

## INTRODUCTION

Chronic myeloid leukemia (CML) is a type of cancer affecting the blood-forming cells of the bone marrow with an incidence of 1.9 cases per 100 000 in the United States.^1^ In approximately 95% of CML cases, a balanced chromosomal translocation between chromosomes 9 and 22 results in a shortened chromosome 22 (called the Philadelphia chromosome) carrying the abnormal *BCR-ABL1* fusion gene.^2,3^ CML is typically diagnosed in the chronic phase (CML-CP) but can progress into the accelerated phase (AP) and eventually the terminal state of blast crisis (BC) in less than 5% of patients, even with medical intervention.^2,4^

Currently, adenosine triphosphate–competitive tyrosine kinase inhibitors (TKIs) that target the BCR-ABL1 oncoprotein are standard of care for CML-CP.^5^ These include the first-generation TKI, imatinib; the second-generation TKIs, dasatinib, nilotinib, bosutinib; and the third-generation TKI, ponatinib. Despite the availability of several TKIs, a proportion of the CML-CP population remains refractory or intolerant to these medications, and these patients cycle through multiple lines of therapy.^5^ Sequential treatment with TKIs may occur with the development of mutations in the *BCR-ABL1* gene, with the T315I mutation conferring resistance to all approved TKIs other than ponatinib.^6^ A lack of standard of care or treatment guidance from guidelines beyond the second line can further complicate CML treatment.^6–8^ As such, treatment failure may occur and is associated with progressively poorer clinical outcomes, especially on or after third-line (3L) therapy.^6,9,10^ Moreover, even with treatment, some patients progress to AP or BC, which is associated with particularly poor clinical outcomes.^4^ For eligible patients with refractory or advanced disease, allogeneic hematopoietic stem cell transplantation (HSCT) may be recommended, but HSCT is suitable for a limited number of patients, has high morbidity and mortality relative to TKI therapy, and is associated with high costs.^5,11^

Prior studies have demonstrated higher healthcare resource utilization (HRU) and healthcare costs associated with treatment failure, but they have focused on the costs during earlier lines of therapy that failed (ie, first-line [1L] and second-line [2L]) or in the year following failure of an earlier line of therapy.^12,13^ There is currently limited available information on the costs of CML care among patients with CML-CP who are receiving later lines (ie, third-line or fourth or later lines [4L+]) of CML therapy, as well as those who progress to AP/BC or who undergo HSCT. Therefore, this study was conducted to describe the treatment patterns, HRU, and costs of CML care from a payer’s perspective, by CML health state, among commercially insured patients in the United States.

## METHODS

### Data Source

Adult patients with CML were identified from the IBM® MarketScan® Commercial Claims and Medicare Supplement Databases from January 1, 2000, to June 30, 2019. These databases include combined claims from over 130 payers from all US census regions, with information on health plan enrollment, demographics, claims for medical services with diagnosis and procedure codes, and claims for pharmacy services. In the commercial claims, all census regions are well represented, although there is a slightly higher representation from the South and North Central (Midwest) regions. The data are de-identified and comply with the confidentiality requirements of the Health Insurance Portability and Accountability Act (HIPAA).

### Study Design and Sample Selection

A retrospective cohort study design was used to address the study objectives. Lines of therapy were identified based on pharmacy and medical claims for CML-CP treatments (ie, the TKIs bosutinib, dasatinib, imatinib, nilotinib, and ponatinib; and omacetaxine). A line of therapy started at the first claim for a given treatment and ended at the first event defined as switch to another CML-CP treatment, initiation of a chemotherapy not listed above for CML-CP, HSCT, treatment discontinuation (ie, treatment gap ≥90 days), end of data availability, or end of continuous health plan enrollment.

### Health State Cohorts

Patients were classified into the following health states: on 3L therapy (hereinafter referred to as CML-CP On Treatment), on 4L+ therapy (CML-CP Post-Discontinuation), and progressed to CML in AP/BC (CML-AP/BC).

Patient selection for the CML-CP On Treatment and CML-CP Post-Discontinuation cohorts was detailed in a previous study (**Supplementary Figure S1**).^14^ Briefly, adult patients were included if they had at least 1 CML diagnosis (*International Classification of Diseases, Ninth Revision, Clinical Modification* [ICD-9-CM] code 205.1x; *International Classification of Diseases, Tenth Revision, Clinical Modification* [ICD-10-CM] code C92.1x), were initiated on 1L therapy for CML-CP indicated for newly diagnosed CML (ie, either bosutinib, dasatinib, imatinib, or nilotinib), and were later initiated on a 3L or 4L+ therapy. For the CML-CP On Treatment cohort, the index date was defined as the date of initiation of 3L therapy, and the follow-up period spanned from the index date until the end of 3L therapy, initiation of a chemotherapy not for CML-CP, HSCT, end of data availability, or continuous health plan enrollment, whichever occurred first. For the CML-CP Post-Discontinuation cohort, the index date was defined as the date of initiation of 4L therapy, and the follow-up period spanned from the index date until end of the last observed line of therapy for CML-CP, initiation of a chemotherapy not for CML-CP, HSCT, end of data availability, or continuous health plan enrollment, whichever occurred first, excluding the treatment-free period between lines.

**Figure 1.**
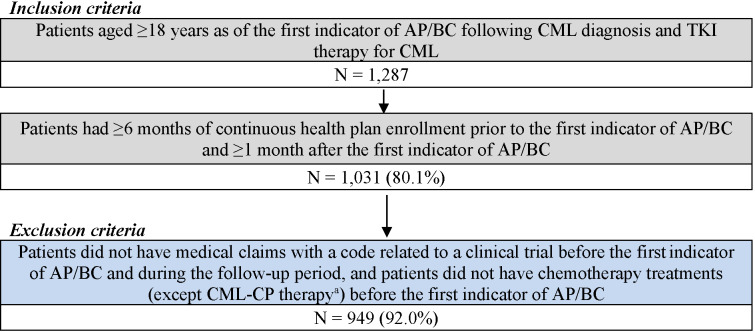
Sample Selection for the CML-AP/BC Cohort Abbreviations: AP/BC, accelerated phase/blast crisis; CML, chronic myeloid leukemia; CP, chronic phase; TKI, tyrosine kinase inhibitor. ^a^CML-CP treatments included imatinib, bosutinib, dasatinib, nilotinib, omacetaxine, or hydroxyurea.

For the CML-AP/BC cohort, patients were included if they had at least 1 CML diagnosis and were treated with any TKI, followed by at least 1 clinical indicator for AP/BC (ie, acute myeloid leukemia [AML; ICD-9-CM code 205.0x; ICD-10-CM code C92.0x] or acute lymphocytic leukemia [ALL; ICD-9-CM code 204.0x; ICD-10-CM code C91.0x] diagnosis or the use of AML/ALL-like chemotherapy [**Supplementary Table S1**]). Additionally, patients were included if they were at least 18 years of age as of the first indicator of AP/BC following CML diagnosis and TKI therapy for CML, had at least 6 months of continuous health plan enrollment prior to the first indicator of AP/BC, and had at least 1 month of continuous health plan enrollment after the first indicator of AP/BC (Figure 1). Patients were excluded if they had medical claims with a code related to a clinical trial and if they underwent chemotherapy (except CML-CP treatments) before the first indicator of AP/ BC. The index date for the AP/BC cohort was defined as the date of the first indicator of AP/BC following CML diagnosis and TKI therapy for CML. The follow-up period spanned from the index date until HSCT, end of data availability, or end of continuous health plan enrollment, whichever occurred first.

Patients in any of the 3 health states above who received HSCT (in an inpatient [IP] setting) were also grouped for analysis in a cohort, hereinafter referred to as the Post-HSCT cohort. The index date for this cohort was defined as the day following the IP discharge date of the first HSCT. The follow-up period spanned from the index date until the end of data availability or the end of continuous health plan enrollment, whichever occurred first.

### Outcomes and Statistical Analysis

The proportion of patients who used each line of therapy (ie, 2L, 3L, 4L, etc) from 2001 to 2019 was assessed among patients with CML-CP who initiated 1L therapy. The prevalence of patients with CML-CP by line of therapy in 2018 (most recent complete year available in commercial claims) was also assessed. Prevalence was defined as the proportion of patients who were managed with 2L or third or later lines (3L+) during the 2018 calendar year (separately), among those who were observed with 1L at some point from 2001 to 2018 in commercial claims with continuous health plan enrollment during that year.

The distribution of CML-CP treatments by patient-month was described by health state and for the Post-HSCT cohort. In addition, the use of hydroxyurea, interferon, and AML/ALL-like chemotherapies was assessed for patients in the CML-AP/BC cohort.

All-cause and CML-related HRU (ie, outpatient [OP] visits, IP days, and emergency department [ED] visits) were summarized using 3-month incidence rates (IR) by health state and for the Post-HSCT cohort. CML-related HRU was defined as medical services associated with a diagnosis of CML or a procedure for omacetaxine in medical claims. For the 3 health states, HRU outcomes were reported excluding medical services with an HSCT-related code (diagnosis or procedure code for HSCT). For the Post-HSCT cohort, HRU was stratified for medical services with and without HSCT-related codes.

All-cause and CML-related event-level costs (ie, cost per OP visit, cost of day per IP stay, and cost per ED visit), excluding medical services with HSCT-related codes, were reported for patients across health states. CML-related costs were also defined using medical services associated with a diagnosis of CML or a procedure for omacetaxine in medical claims. Event-level costs for OP visits and IP days with a HSCT-related code were also reported for the Post-HSCT cohort. Event-level costs were also reported for the first HSCT observed in an IP setting and for terminal care costs, which included any medical and pharmacy costs incurred in the 3-month period preceding an IP discharge date with a mortality code. Costs were summarized using means, medians, and SD and reported from a payer’s perspective (including the paid amount by the health plan and coordination of benefits) and reported in 2019 USD.

## RESULTS

### Sample Selection

Among the 3234 patients identified with 1L therapy, 954 (29.5%) patients also received 2L therapy, 296 (9.2%) received 3L therapy, and 83 (2.6%) subsequently received 4L therapy for CML between 2001 and 2019 (**Supplementary Figure S2**). In 2018, the last complete year of data, the prevalence of 2L therapy was 21.2% and 3L+ was 9.7%.

Among the 296 patients in the CML-CP On Treatment cohort (patient-months = 4620), median age at 3L initiation was 58 years; 49.7% were female. Among the 83 patients in the CML-CP Post-Discontinuation cohort (patient-months = 1644), median age at initiation of 4L therapy was 62 years; 48.2% were female. Further characterization of these patients was published previously.^14^

Among the 949 patients in the CML-AP/BC cohort (patient-months = 25 593), median age at the first AP/BC indicator was 56 years; 45.8% were female. A total of 846 patients (89.2%) had a diagnosis of AML/ALL following CML diagnosis and TKI use. Additionally, 198 patients (20.9%) used AML/ALL-like chemotherapies, the most common of which were methotrexate (29.8%), cytarabine (20.2%), and vincristine (9.1%). During the follow-up period, 740 patients (78.0%) were treated with TKIs.

Among the 97 patients in the Post-HSCT cohort (patient-months = 2471), median age at HSCT was 49 years; 40.2% were female. Forty patients (41.2%) were treated with TKIs (patient-months = 300) during the follow-up period, and TKI therapy was resumed within the first 3 months after HSCT for the majority of patients (57.5%).

### Treatments by Health State

In the CML-CP On Treatment cohort, second-generation TKIs (ie, nilotinib, dasatinib, and bosutinib) were most commonly used (69.1% of patient-months), followed by the first-generation TKI imatinib (27.8% of patient-months), and the third-generation TKI ponatinib (3.1% of patient-months; Table 1). Similarly, in the CML-CP Post-Discontinuation cohort, second-generation TKIs were most commonly used (59.1% of patient-months), followed by imatinib (32.7% of patient-months) and ponatinib (7.0% of patient-months; Table 1). In the CML-AP/BC cohort, imatinib was most commonly used (33.5% of patient-months), followed by second-generation TKIs (20.2% of patient-months); however, no treatment was observed in 42.5% of patient-months. Following HSCT, 40 patients used TKIs (41.2%), with second-generation TKIs (49.3% of patient-months) most commonly used, followed by imatinib (22.0% of patient-months), and ponatinib (19.1% of patient-months). Among these patients, the mean time from HSCT to initiation of TKI was 6.8 months (median 2.5 months).

**Table 1.** Distribution of CML Treatments by Health State and for the Post-HSCT Cohort

	**CML-CP On Treatment (4620 Patient-Months), %**	**CML-CP Post-Discontinuation (1644 Patient-Months), %**	**CML-AP/BC (25 593 Patient-Months), %**	**Post-HSCT^a^ (300 Patient-Months), %**
TKI alone				
Nilotinib	40.1	9.3	9.4	22.0
Imatinib	27.8	32.7	33.5	28.6
Dasatinib	20.8	26.5	9.9	18.8
Bosutinib	8.2	23.3	0.9	8.6
Ponatinib	3.1	7.0	0.6	19.1
Omacetaxine	0.0	1.1	0.0	0.0
TKI and AML/ALL-like chemotherapy	—	—	1.2	3.0
AML/ALL-like chemotherapy alone	—	—	1.2	—
Interferon alone	—	—	0.0	—
Hydroxyurea alone	—	—	0.8	—
No treatment	—	—	42.5	—

Abbreviations: ALL, acute lymphocytic leukemia; AML, acute myeloid leukemia; AP/BC, accelerated phase/blast crisis; CML-CP, chronic myeloid leukemia-chronic phase; HSCT, hematopoietic stem cell transplantation; TKI, tyrosine kinase inhibitor.

^a^Treatments reported among the 40 patients observed with TKI use post-HSCT.

### Healthcare Resource Utilization

**Figure 2.**
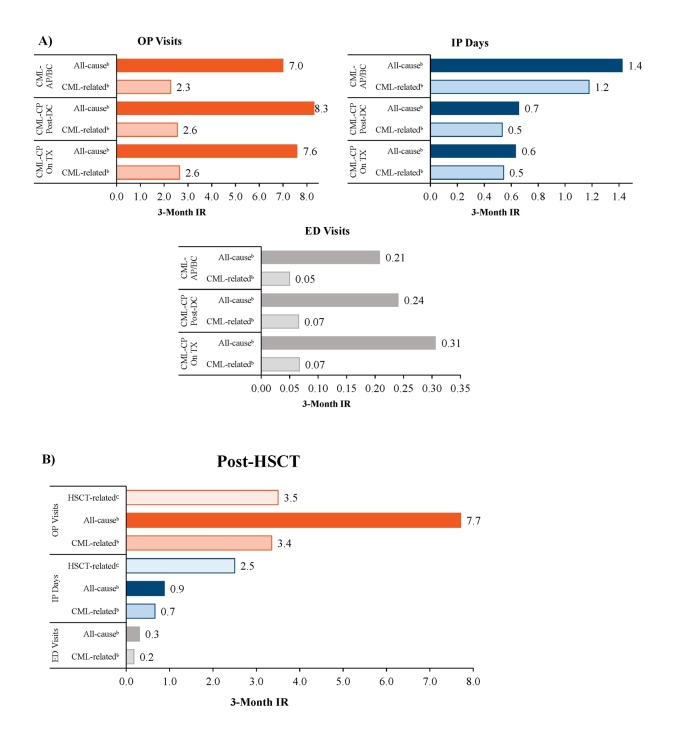
Healthcare Resource Utilization Among the CML-CP On Treatment, CML-CP Post-Discontinuation, and CML-AP/ BC Cohorts **(A)** and the Post-HSCT Cohort **(B)**^a^ Abbreviations: AP/BC, accelerated phase/blast crisis; CML-CP, chronic myeloid leukemia–chronic phase; DC, discontinuation; ED, emergency department; HSCT, hematopoietic stem cell transplantation; IP, inpatient; IR, incidence rate; OP, outpatient; TX, treatment. ^a^Patient-months: CML-CP On Treatment = 4620; CML-CP Post-Discontinuation = 1644; CML-AP/BC = 25 593; Post-HSCT = 2471. ^b^IRs excluding medical services related to HSCT. ^c^IRs for medical services with HSCT-related codes.

Patients in the CML-CP On Treatment, CML-CP Post-Discontinuation, and CML-AP/BC cohorts had 7.0 to 8.3 all-cause OP visits per 3 months, with 2.3 to 2.6 of these being CML-related OP visits (Figure 2A). Patients in the CML-AP/BC cohort had 1.4 all-cause IP days (1.2 CML-related) per 3 months, while patients in the CML-CP On Treatment and CML-CP Post-Discontinuation cohorts had 0.6 to 0.7 all-cause IP days (0.5 CML-related) per 3 months. Patients in the CML-CP On Treatment, CML-CP Post-Discontinuation, and CML-AP/BC cohorts had 0.21 to 0.31 all-cause ED visits per 3 months, with 0.05 to 0.07 of these being CML-related ED visits.

Patients in the Post-HSCT cohort had 7.7 all-cause OP visits (3.4 CML-related) and 3.5 HSCT-related OP visits, 0.9 all-cause IP days (0.7 CML-related) and 2.5 HSCT-related IP days, and 0.3 ED visits (0.2 CML-related) per 3 months (Figure 2B).

### Healthcare Costs

Across all health states, on average, each all-cause OP visit cost $597, each all-cause IP day cost $5892, and each all-cause ED visit cost $1923 (Table 2). The first HSCT in an IP setting had a mean cost of $352 333 per patient and terminal care costs totaled $107 013 per patient. After HSCT, on average, OP visits with a HSCT-related code cost $2280 and IP with a HSCT-related code cost $23 584 per day.

**Table 2.** Healthcare Costs at the Event Level

	**Events (n)**	**Payers’ Cost per Event (2019 USD), Mean ± SD [Median]**
OP visits		
OP visits with HSCT-related codes^a^	2852	2280 ± 3664 [961]
All-cause^b,c^	76 043	597 ± 2770 [135]
CML-related	24 916	867 ± 3875 [188]
IP (daily cost)		
IP days with HSCT-related codes^a^	2029	23 584 ± 202 929 [4839]
All-cause^b,c^	13 497	5892 ± 6997 [3995]
CML-related	11 177	5900 ± 6833 [4107]
ED visits		
All-cause^b,c^	2382	1923 ± 3708 [836]
CML-related	564	2637 ± 5036 [1372]
First HSCT in an IP setting^a,d^	97	352 333 ± 333 171 [243 193]
Terminal care costs^d^	38	107 013 ± 89 845 [82 667]

Abbreviations: CML, chronic myeloid leukemia; ED, emergency department; HSCT, hematopoietic stem cell transplantation; IP, inpatient; OP, outpatient; USD, United States dollars.

^a^Events and costs reported post-HSCT.

^b^Events and costs reported from the CML-CP On Treatment, CML-CP Post-Discontinuation, and CML-AP/BC cohorts.

^c^Costs excluding medical services with HSCT-related codes.

^d^Costs reported at the patient level.

## DISCUSSION

In this retrospective cohort study of commercially insured patients with CML who received later lines of therapy or who progressed to AP/BC, rates of HRU and medical costs were high. To our knowledge, this is the first study to assess HRU and costs across various CML health states, including those with 3L, 4L+, or in AP/BC in any setting. The study findings highlight an unmet need for CML patients in later lines of therapy and are consistent with previous studies that have reported increasing HRU and cost outcomes among patients with CML and 2L therapy failure relative to 1L,^13^ as well as healthcare costs associated with treatment failure overall.^12^ For example, in a US claims-based study by McGarry et al,^13^ patients with 2L treatment failure incurred higher HRU and $20 957 in 2012 USD ($25 148 in 2019 USD) higher total healthcare costs in the 1 year post-TKI treatment failure than those with 1L treatment failure. Increasing mean healthcare costs were observed from 1L to 2L to 3L treatment failure, at $181 029 in 2012 USD ($217 234 in 2019 USD) per treatment episode (81.1% from medical services) for patients with 3L TKI failure, suggesting an increasing burden associated with treatment failure with each subsequent line of therapy.

In the current study, all-cause IP utilization rates, excluding services with an HSCT-related code, were particularly high among patients with CML who progressed to AP/BC, with a mean 3-month IR of 1.4 days. Substantial medical costs were also observed, with mean daily IP costs of $5982. Although HSCT remains a viable treatment option among patients with CML-CP or AP/BC who meet the specific eligibility criteria, HSCT was associated with significant medical costs ($352 333 at the event-level) and continues to be a prohibitively expensive treatment option, especially considering the cost of post-transplantation care.^15^ Treatment options among patients in AP/BC were also observed to be suboptimal, with imatinib treatment occurring in approximately one-third of patient-months, and almost half of patient-months were found to not have any standard CML treatments, including TKIs or other chemotherapies.

Studies have reported worse clinical outcomes associated with multiple lines of TKI use, including decreased probability of response, earlier time to failure, worse overall survival, and the development of resistant mutation variants.^6,13^ Real-world clinical studies of treatment response among patients with CML on 3L therapy with second-generation TKIs, who failed 2 prior TKIs, have reported complete cytogenetic response rates that ranged between 6% and 21%.^10,16–18^ An academic-based, single-center, retrospective clinical study of patients with CML-CP reported overall survival among patients treated with TKIs who did not respond to more than 1 line of therapy was between 13% to 45% lower, as compared with those who responded to a single line of therapy.^19^ Our findings of elevated HRU and healthcare costs in later lines may be attributed to the poorer clinical outcomes observed among these patients, but unfortunately information on assessment of molecular or cytogenetic response was not available in the claims data source.

Although progression to AP/BC does not occur frequently among patients with CML-CP, with national multicenter registries reporting incidences of less than 5%,^4^ disease progression is not only associated with poor outcomes but also with substantial healthcare costs. Data from a US claims-based study by Jabbour et al^20^ demonstrated that patients with CML who progressed to BP incurred a significantly greater mean number of hospitalizations, longer mean length of hospital stay, more OP visits, and more prescription claims than those without progression. This translated to $270 925 in 2015 USD ($302 081 in 2019 USD) higher mean 1-year total all-cause healthcare costs for patients with vs without progression. The current study findings complement the proven costly burden associated with treatment failure in earlier lines by adding important insight among patients with 3L or 4L+ of treatment and progression to AP/BC.

Parallel to the large economic burden observed with later lines of therapy and CML progression, HSCT was also associated with considerable costs in our analysis. Among 97 procedures observed in an IP setting, the mean event-level cost of a HSCT was $352 333 (median, $243 193). Among patients with CML-CP who experienced a HSCT, the mean daily cost of an IP stay was 4 times higher than the daily cost observed among all patients on later lines or who progress to AP/BC. Although there is little information on HSCT-associated costs in later lines of therapy for patients with CML-CP, results from the current study are consistent with a previous study that reported annual HCST costs in claims 1 year post–TKI treatment failure, which ranged between $210 612 and $243 435 in 2012 USD ($252 734-$292 122 in 2019 USD), depending on the line of treatment.^13^

Treatment patterns observed by health state demonstrated high proportions of imatinib use, which were observed from a range of 27.8% of patient-months among patients in the CML-CP On Treatment cohort to 33.5% of patient-months among patients in the AP/BC cohort. This finding may be partially explained by the study design, with initial TKI therapy starting in 2001 and the first new second-generation TKI approved for treatment of CML among patients who were refractory or intolerant to imatinib only approved in 2006.^21^ As a result, many patients only had access to imatinib as a treatment option for CML-CP. In addition, clinical practice guidelines for the management of CML provide limited guidance on the use of specific TKI therapies beyond 2L to guide treatment decisions.^6–8^ As a result, some clinicians may have reverted back to imatinib after treatment failure with a second-generation TKI. Indeed, a previous study that has examined the CML-CP On Treatment cohort, the combination of imatinib (1L) → dasatinib (2L) → imatinib (3L) was one of the most frequent treatment sequences observed (8.8%),^14^ potentially illustrating the effects of this lack of treatment guidance. Furthermore, among the 97 patients in the Post-HSCT cohort, 41.2% resumed TKI therapy after a HSCT despite the lack of clear guideline recommendations on TKI use for these patients.

There is little information on the treatment patterns of patients with CML who have progressed to AP/BC in the United States. In this study, a large proportion of patients in AP/BC had no treatments observed (42.5% of patient-months). This finding may represent a lack of effective and tolerable treatment options for this population. Indeed, advanced phases of CML are typically resistant to TKI treatment and have a worse prognosis, with many patients dying from infection or bleeding complications.^4^ In addition, some patients, particularly those in BC, who experience survival less than 1 year following diagnosis, may be directly sent to palliative care because of terminal illness and thus may not use TKIs or other treatments like hydroxyurea.^7^ Adverse events related to TKIs, including cytopenia and pleural effusions, may also occur frequently among patients with CML who progressed to AP/BC, and thus gaps in therapy may be observed to manage these adverse events.^22,23^

### Limitations

The results of the current study should be interpreted in light of several limitations. First, laboratory test results, such as cytogenetic and molecular responses, as well as mutation testing results, were not available in MarketScan® database. As a result, we were unable to ascertain reasons for ending the line of therapy (ie, intolerance, lack of efficacy, or resistance to TKI). Second, existing diagnosis codes for CML do not distinguish between CP and AP/BC. It may be possible that some patients in the CML-CP On Treatment and CML-CP Post-Discontinuation health states may have been in AP/BC, but this is likely to be minimal and not impact the study findings given the low rates of disease progression. Third, an algorithm was used to identify AP/BC patients based on the use of indicators observed in claims. These indicators included the use of AML/ALL-like chemotherapies, which may also be used to treat additional malignancies. Fourth, given that terminal care costs were identified based on the observed admission discharge status for death, the total number of deaths may be underestimated and costs overestimated since all patients required an IP admission. Fifth, these study findings among patients with CML with access to commercial insurance or Medicare Supplemental plans may not be generalizable to the overall CML population in the United States. In addition, the proportion of patients in later lines of therapy may have been underestimated due to right data censoring (ie, end of data availability or end of continuous health plan enrollment). Finally, pharmacy claims for a filled prescription do not guarantee the actual consumption of the medication by the patient and therefore some patients may have ended a line of treatment even before the end was identified in claims. Despite these limitations, administrative claims databases remain an important source to assess HRU and costs of CML management since it includes diagnosis and procedure codes that are recorded for reimbursement purposes.

## CONCLUSIONS

This study fills an important gap in knowledge by using claims data to characterize patients with CML-CP receiving later lines of therapy or progressing to AP/BC, who have not been well studied. HRU and cost of CML care in the United States is substantial among patients with CML who are in 3L, 4L+ or progressed to AP/BC, suggesting that the disease is associated with an important economic and clinical burden. Healthcare resource utilization was observed to increase from 3L to 4L+, and the incidence of IP days was particularly high for those who progressed to AP/BC. Further research is warranted to determine if better treatment options for patients with CML-CP undergoing later lines of therapy or progressing to AP/BC may help to alleviate the burden.

### Disclosures

E.L.A. reports personal fees from Novartis, grants and personal fees from Takeda, and personal fees from Bristol Myers Squibb. R.M. is an employee of Novartis Pharmaceuticals Corporation, which funded the development and conduct of this study and manuscript. D.L.V., C.R., and A.G. are employees of Analysis Group, Inc, a consulting company that has provided paid consulting services to Novartis Pharmaceuticals Corporation.

### Author Contributions

D.L.V., C.R., and A.G. contributed to study conception and design, collection and assembly of data, and data analysis and interpretation. E.L.A. and R.M. contributed to study conception and design, data analysis and interpretation. All authors reviewed and approved the final content of this manuscript.

### Prior Presentations

Part of the material in this manuscript was presented at the Virtual International Society for Pharmacoeconomics and Outcomes Research (ISPOR) Annual Meeting, May 17-20, 2021, as a poster presentation.

## Supplementary Material

Online Supplementary Material
