# Supplementary material for: Chronic Myeloid Leukemia: Part II—Cost of Care Among Patients in Advanced Phases or Later Lines of Therapy in Chronic Phase in the United States from a Commercial Perspective
Source: J Health Econ Outcomes Res. 2022 Aug 4;9(2):30–6. doi: 10.36469/001c.36976 (PMC9353133; doi:10.36469/001c.36976)
Supplement: Online Supplementary Material [file jheor_2022_9_2_36976_95913.pdf]

Journal of Health Economics and Outcomes Research

Online Supplementary Material

Chronic Myeloid Leukemia: Part II—Cost of Care Among Patients in Advanced Phases or Later Lines of Therapy in Chronic Phase in the United States from a Commercial Perspective. *JHEOR*. 2022;9(2):30-36. doi:10.36469/jheor.2022.36976

Table S1: List of AML-like or ALL-like Chemotherapies

Figure S1: Sample Selection for the CML-CP On Treatment and CML-CP Post-discontinuation Cohorts

Figure S2: Proportion of Patients by Line of Therapy (2001-2019)

This supplementary material has been provided by the authors to give readers additional information about their work.

This is an open-access article distributed under the terms of the Creative Commons Attribution 4.0 International License (CCBY-4.0). View this license's legal deed at http://creativecommons.org/licenses/by/4.0 and legal code at http://creativecommons.org/licenses/by/4.0/legalcode for more information.

Table S1. List of AML-like or ALL-like Chemotherapies

Azacitidine
Blinatumomab
Cyclophosphamide
Cytarabine
Daunorubicin
Decitabine
Doxorubicin
Gemtuzumab
Idarubicin
Inotuzumab
Mercaptopurine
Methotrexate
Pegaspargase
Vincristine

Abbreviations: ALL, acute lymphocytic leukemia; AML, acute myeloid leukemia.

Figure S1. Sample Selection for the CML-CP On Treatment and CML-CP Post-discontinuation Cohorts

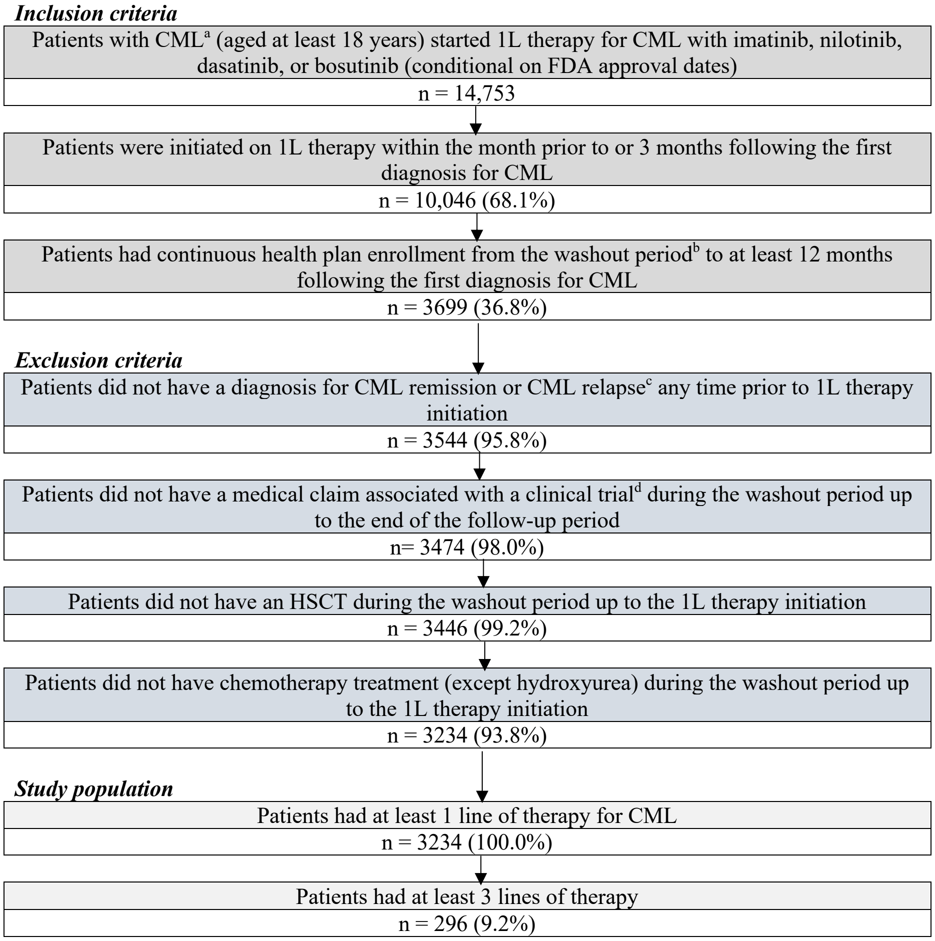

Abbreviations: 1L, first-line; 3L, third-line; CML-CP, chronic myeloid leukemia in chronic phase; FDA, Food and Drug Administration; HSCT, hematopoietic stem cell transplant; Ph+, Philadelphia chromosome–positive

[a]Patients had at least 1 diagnosis for CML (ICD-9-CM code 205.1x, ICD-10-CM code C92.1x), with first CML diagnosis observed in claims on or after May 10, 2001, the date of FDA approval for imatinib.

[b]The washout period was defined as a period of at least 6 months prior to 1L therapy initiation for CML.

[c]CML remission was defined using ICD-9-CM code 205.11 or ICD-10-CM code C92.11, and CML relapse was defined using ICD-9-CM code 205.12 or ICD-10-CM code C92.12.

Figure S2. Proportion of Patients by Line of Therapy (2001-2019)

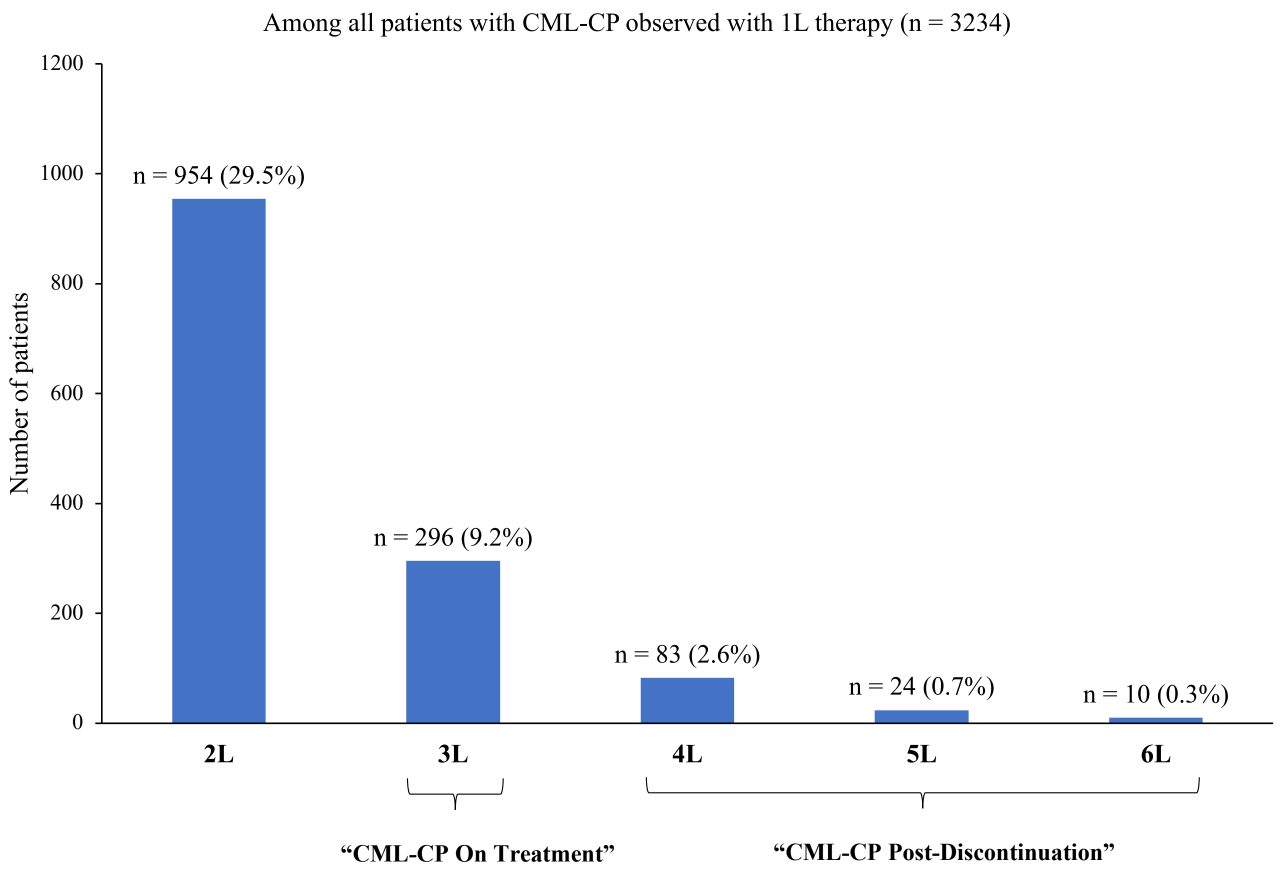

Abbreviation: CML-CP, chronic myeloid leukemia–chronic phase.
